# Microbial dysbiosis in periodontitis and peri-implantitis: pathogenesis, immune responses, and therapeutic

**DOI:** 10.3389/fcimb.2025.1517154

**Published:** 2025-02-11

**Authors:** Ziwei Cui, Peng Wang, Weiyue Gao

**Affiliations:** Stomatology Center, Gansu Provincial Hospital, Lanzhou, Gansu, China

**Keywords:** periodontitis, peri-implantitis, oral microorganisms, immune homeostasis, microbial dysbiosis

## Abstract

The oral microbiome comprises over 700 distinct species, forming complex biofilms essential for maintaining oral and systemic health. When the microbial homeostasis in the periodontium is disrupted, pathogens within the biofilm can cause periodontitis and peri-implantitis, inducing host immune responses. Understanding the role of microbial communities and the immune mechanisms in oral health and disease is crucial for developing improved preventive, diagnostic and therapeutic strategies. However, many questions remain about how changes in bacterial populations contribute to the development and progression of these conditions. An electronic and manual literature search was conducted using PubMed, Excerpta Medica, Frontiers Reports and the Wiley Online Library databases for relevant articles. Data from these publications were extracted and the overall findings were summarized in a narrative manner. The variations in microbial communities and immune responses of periodontitis and peri-implantitis are explored. Dysbiosis of the subgingival microbiome—characterized by an increase in pathogenic bacteria such as *Porphyromonas gingivalis*, *Tannerella forsythia*, and *Aggregatibacter actinomycetemcomitans*—plays a pivotal role in the initiation and progression of periodontitis. As for peri-implantitis, alterations include a higher abundance of opportunistic pathogens and reduced microbial diversity around implants. Moreover, oral dysbiosis potentially influencing systemic health through immune-mediated pathways. Regional immunity of periodontium involving neutrophils, T helper cells-17, and immune-related cytokines is crucial for maintaining periodontal homeostasis and responding to microbial imbalances. Additionally, the impact of non-mechanical treatments—such as probiotics and laser therapy—on the oral microbiome is discussed, demonstrating their potential in managing microbial dysbiosis. These findings underscore that bacterial dysbiosis is a central factor in the development of periodontitis and peri-implantitis. Maintaining microbial balance is essential for preventing these diseases, and interventions targeting the microbiome could enhance treatment outcomes. Strategies focusing on controlling pathogenic bacteria, modulating immune responses, and promoting tissue regeneration are key to restoring periodontal stability. Further research is needed to clarify the mechanisms underlying the transition from peri-implant mucositis to peri-implantitis and to optimize prevention and treatment approaches, considering the complex interactions between the microbiome and host immunity.

## 1 Introduction

The composition of the oral microbiome can vary greatly between individuals and even within the same individual, depending on factors such as age, lifestyle and genetic background (Di Stefano et al., 2022). Typically, hundreds of microbial species coexist in a mutually dependent and harmonious relationship with the host, forming a large commensal. The environment of the oral cavity, including optimal temperature, humidity and the abundant nutrient sources creates ideal conditions for the survival of bacteria, fungi and viruses. Meanwhile, the stable microbial community in the oral cavity plays a crucial role in maintaining the body’s internal homeostasis. For instance, niches in the subgingival regions of the oral cavity provide an ideal habitat for certain anaerobic proteolytic bacteria (Socransky and Haffajee, 2005). The commensal serves as a natural defense barrier against external or opportunistic pathogens. The oral microbiome acquires nutrients from food residues, with their metabolic byproducts absorbed by the host, supporting cardiovascular health and promoting the mineralization of hard tissues (Kapil et al., 2010). Therefore, the significance of the oral microbiota to human health is comparable to that of the gut microbiota. Given the unique ecosystem of the oral cavity, only about half of the microbial species can be cultured *in vitro* (Aas et al., 2005), and a substantial proportion of the oral microbiome remains uncharacterized, which presents challenges to comprehensive understanding the human microbiome and describing its roles in health and disease.

Data from the Human Microbiome Project (HMP) reveal that the oral microbiome comprises over 700 distinct species. These microorganisms seldom exist in a planktonic state. Instead, they aggregate within various microenvironments, embedded in the extracellular polymeric matrix (EPM) (Bowen et al., 2018), forming biofilm communities, which are highly organized in structure and function (Arweiler and Netuschil, 2016). The EPM supplies essential substances such as water, polysaccharides, proteins, lipids and DNA, which support microbial colonization and stabilize the microbial community. Moreover, EPM facilitates the adherence of the biofilm to host tissues, enhancing both interspecies and intraspecies interactions while providing protection from host immune defenses and antimicrobial agents (Cugini et al., 2019). Gram-positive and Gram-negative bacteria within oral biofilms exchange information via quorum sensing, a mechanism through which bacteria secrete diffusible signaling molecules. This process coordinates a range of physiological and pathological activities for the microbiome, such as making biofilm formation and growth, adapting to environmental changes in oral cavity, competing for superiority against potential rivals, and expressing virulence factors that enable pathogens to cause disease (Shao and Demuth, 2010). These biofilms could proliferate on the mineralized surfaces of dental enamel, leading to periodontitis; or adhere to the surfaces of titanium implants, contributing to both peri-implant mucositis and peri-implantitis. Alterations in the oral microbiota are evident in various diseases, impacting both host metabolism and immune responses. As microbial ecological balance is disrupted, pathogenic bacteria outcompete commensal species and result in a range of oral diseases, including dental caries, periodontitis, oral candidiasis and potentially more severe infections, which are even associated with herpes simplex virus co-infection. The complex anatomical and structural organization of periodontal tissues makes them well-suited for microbial colonization.

Periodontitis and peri-implantitis are infectious diseases that are both initiated by pathogenic microorganisms in dental plaque and perpetuated by multiple factors, including the host immune response (Papapanou et al., 2018). Dysbiosis of the subgingival microbiome along with the emergence of periodontitis-associated pathogens plays a pivotal role in the initiation and progression of periodontitis (Mombelli, 2018). Currently, 16S Ribosomal RNA (16S rRNA) sequencing, metagenomics, and metabolomics are widely used to analyze the biological information of microbial communities (Belibasakis and Manoil, 2021), and over 400 bacterial species have been identified in subgingival plaque using these culture-independent techniques (Di Stefano et al., 2022). Bacterial balance and host immune homeostasis are essential for maintaining the health of periodontal and peri-implant tissues. Alterations in the composition and function of the oral microbiome can change symbiotic interactions between microbial communities and the host, ultimately impacting both oral and systemic health. Similarly, the peri-implant environment shapes the resident microbiota. Understanding the ecological triggers of microbial pathogenicity is crucial for developing improved preventive, diagnostic and therapeutic strategies. However, many questions remain to be explored. Therefore, this review focuses on the role of microbial communities in oral health, the relationship between the microbiome and the health of periodontal and peri-implant tissues, and provids a comprehensive overview of how bacterial population changes contribute to the development and progression of periodontitis and peri-implantitis.

## 2 Methods

Electronic and manual search was conducted for each of the addressed subjects. The PubMed database of the US National Library of Medicine, the Excerpta Medica database of Elsevier, and the Wiley online library were screened for relevant articles (i.e. consensus reports, reviews, experimental studies in animals and humans/observational studies, randomized/controlled clinical studies, meta‐analyses). Data from publications were extracted and overall findings were summarized in a narrative manner.

## 3 Observations and discussion

### 3.1 Colonization changes of subgingival microbiota

In healthy periodontal tissues, a wide variety of oral microorganisms coexist to collectively maintain microenvironmental homeostasis. Approximately ten predominant genera contribute to this balance, each fulfilling distinct roles (Roberts and Darveau, 2015). *Streptococcus* acts as a pioneer species in biofilm formation, initially adhering to teeth surfaces by producing extracellular polysaccharides via glycosyltransferases, which in turn enable the attachment of other microorganisms. Through the production of organic acids, such as lactic acid, *Streptococcus* helps regulate the local pH environment (Boisen et al., 2021). Additionally, it produces antimicrobial substances like hydrogen peroxide, which inhibit pathogenic bacteria, including cariogenic and periodontal pathogens (Sumioka et al., 2017; Baty et al., 2022). Beyond these functions, *Streptococcus* also interacts with the host immune system to strengthen mucosal barrier integrity and prevent the overactivation of inflammatory responses (Ingendoh-Tsakmakidis et al., 2020). *Veillonella* acts as a secondary colonizer, establishing a mutualistic relationship with *Streptococcus*. By metabolizing lactic acid produced by *Streptococcus* as the carbon source, *Veillonella* supports other microbial species within the biofilm, helps regulate local acidity, and indirectly protects tooth enamel from erosion (Violant et al., 2014; Zhang and Huang, 2023). *Actinomyces* works in synergy with *Streptococcus* to establish a mature biofilm structure. By degrading complex carbohydrates, it produces acidic metabolites that other microbial species utilize (Liy et al., 2008). However, *Actinomyces* displays a dual nature: it helps maintain ecological balance under healthy conditions but, when overabundant, can contribute to root caries or chronic periodontitis (Meuric et al., 2017; Del Pilar Angarita-Díaz et al., 2024). *Corynebacterium* serves as a structural backbone in mature biofilms, providing physical attachment points for other microbial species (Rogers et al., 2011). Under healthy conditions, it inhibits the overgrowth of pathogenic bacteria (Meuric et al., 2017). *Rothia*, recognized as a marker of oral health, is strongly linked to a balanced and healthy oral environment through its special nitrate reduction effect. It contributes to the breakdown of carbohydrates and proteins, supporting the metabolic exchange within microbial communities. Moreover, it may interact with the host immune system to suppress overactive inflammatory responses (Fatahi-Bafghi, 2021; Mazurel et al., 2023). *Neisseria* plays a role in the initial formation of periodontal biofilms. It also converts salivary nitrate into nitrite, contributing to the host’s redox homeostasis and vascular function modulation (Pignatelli et al., 2020; Rosier et al., 2022). In a healthy oral environment, it supports the ecological stability of the microbial community. *Capnocytophaga* attaches to the tooth surface by producing extracellular polysaccharides, enhancing the stability of biofilms. It interacts with host immune cells, potentially modulating local inflammation through the secretion of regulatory factors (Bolton et al., 1985; Idate et al., 2020). This bacterium exhibits a dual nature: at low abundance, it contributes to maintaining a healthy microbial balance, while at high abundance, it is often associated with periodontal disease (Ciantar et al., 2005). *Haemophilus* works synergistically with *Streptococcus* and *Actinomyces* to maintain the stability of the oral ecosystem (Del Pilar Angarita-Díaz et al., 2024). *Fusobacterium* acts as a “bridge” species within the biofilm, linking early colonizers and late-stage pathogenic bacteria by facilitating interspecies connections (Horiuchi et al., 2020). It plays a role in modulating host immune responses, thereby promoting the dynamic equilibrium of the biofilm. Moreover, *Fusobacterium* contributes to the degradation of complex organic compounds, supporting the coexistence of various microbial communities (Li et al., 2020). *Prevotella* breaks down complex carbohydrates and releases organic acids that serve as an energy source for other microbial species (Gellman et al., 2023). This genus also demonstrates a dual nature: at low abundance, it contributes to maintaining a healthy microbial ecosystem, but at higher abundance, it is frequently linked to the development of periodontal diseases (Sharma et al., 2022).

The stable ecological environment of the gingival sulcus can be disrupted by changes in the host or microbiome. The salivary flow redistributes the various cytokines and immunoglobulins produced by the immunocytes, and then will influence microbial colonization. The proliferation of certain bacteria, such as *Porphyromonas gingivalis*, could modify the nutrient environment at the colonization site, disrupting the symbiotic balance between the microbiota and host (Hajishengallis, 2014). Additionally, regulation of bacteriophage impacts the composition and abundance of microbial communities (Wang et al., 2013). Consequently, host-driven species-specific inhibition and microbial competition will contribute to potential pathogen colonization and enhance the pathogenicity of the microbiome.

As a foreign object, the implant creates an initial pocket after placement, which rapidly attracts the attachment of the salivary pellicle to its surface. Within 30 minutes, the adhesion and colonization of early colonizers can be observed in the implant sulcus, and over the next two weeks, the early microbes develop into an organized biofilm community within the peri-implant space (Belibasakis et al., 2015), offering surface receptors for the attachment and proliferation of late colonizers (Kolenbrander et al., 2010). The most common taxonomic groups found in the subgingival plaque samples from dental implants at 4 weeks were *Streptococcus* (20%), *Fusobacterium* (16%), *Neisseria* (12%) and *Prevotella* (9%), while the most common taxa in the subgingival plaque samples from dental implants changed at 12 weeks were *Streptococcus* (22%), *Fusobacterium* (16%), *Neisseria* (8%) and *Rothia* (7%). Meanwhile, within the subgingival plaque samples from control teeth at 4 weeks, the most common taxonomic groups were *Streptococcus* (22%), *Fusobacterium* (17%), *Neisseria* (7%) and *Prevotella* (6%), while *Fusobacterium* (20%), *Streptococcus* (18%), *Prevotella* (8%) and *Veillonella* (6%) were more common at 12 weeks. Initially, periodontal bacteria such as *Corynebacterium*, *Actinomyces*, *Selenomonas*, and *Campylobacter* are difficult to detect around the implant, but their abundance steadily rises after the 12th week of post-implantation (Dabdoub et al., 2013; Schaumann et al., 2014). In addition, in the fourth week, the expression of granulocyte-macrophage colony-stimulating factor (GM-CSF) was significantly increased in both gingival crevicular fluid (GCF) and peri-implant crevicular fluid (PICF), not only that, higher interleukin-10 (IL-10) levels were detected in samples from failed implants during this period (Payne et al., 2017). The microscopic evidence indicates that the 4th to 12th week post-implantation is a critical risk period for implant failure.

### 3.2 Alterations of the oral microbiota in periodontitis

**Figure 1:**
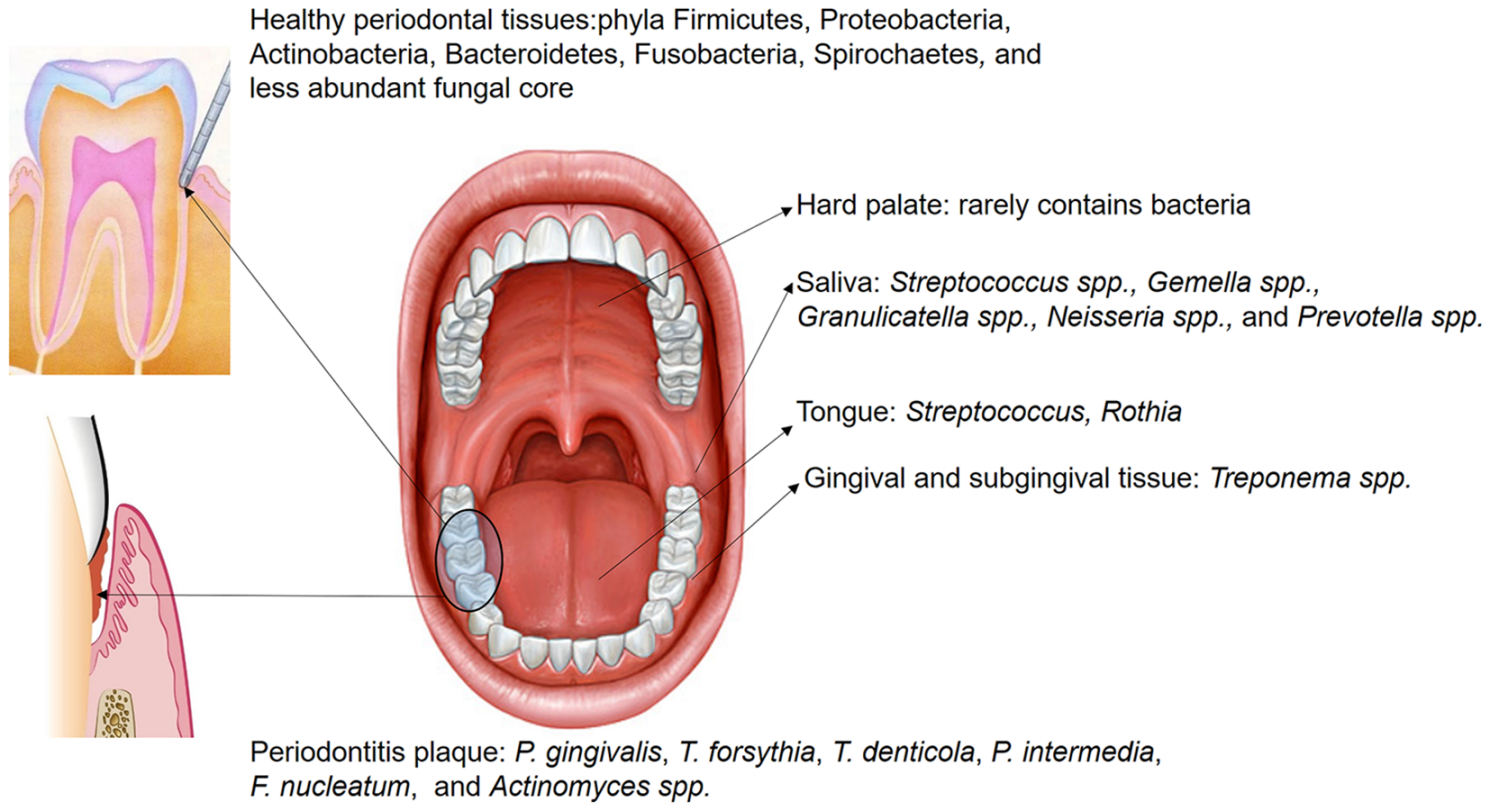
The distribution of microbiota within different oral regions exhibits overlap across sites; however, certain bacteria are predominantly localized in specific areas. Saliva hosts numerous non-pathogenic microorganisms, including *Streptococcus* spp., *Gemella* spp., *Granulicatella* spp., *Neisseria* spp., and *Prevotella* spp. In the healthy gingival sulcus, the dominant phyla are Firmicutes, Proteobacteria, Actinobacteria, Bacteroidetes, Fusobacteria, Spirochaetes, and a smaller core of fungal species. Conversely, in subgingival plaque of periodontitis, the abundance of pathogenic species, such as *P. gingivalis*, *T. forsythia*, *T. denticola*, *P. intermedia*, *F. nucleatum*, and *Actinomyces* spp., is significantly increased.

Periodontal disease has emerged as a major public health concern that affects an estimated 20% to 50% of the global population, with its prevalence anticipated to rise in the coming years. Microorganisms play a pivotal role in regulating the acid-base balance within the oral cavity (Struzycka, 2014; Gupta et al., 2024). When compared to healthy controls, patients with chronic gingivitis display a more alkaline salivary pH, whereas those with chronic periodontitis tend to have a more acidic pH, indicating that pH variation in the oral cavity probably depends on the severity of periodontal inflammation (Baliga et al., 2013). This view leads to changes in the characterization of microbial diversity and alterations in the composition of the microbiota. In the healthy oral cavity, the predominant microbial species include *Streptococcus*, *Prevotella*, *Haemophilus*, *Fusobacterium*, and *Veillonella*. The composition and abundance of these microorganisms vary significantly across distinct anatomical regions, including saliva, buccal mucosa, hard palate, palatine tonsils, subgingival or supragingival areas, pharynx and dorsal surface of the tongue (Maruyama et al., 2014). In healthy periodontal tissues, the predominant microbial community consists of Gram-positive facultative anaerobes, such as *Streptococcus* and *Actinomyces*. Among them, *Proteobacteria* are especially abundant, forming symbiotic associations with several species within the *Firmicutes* phylum. These bacteria do not typically cause inflammation but rather exist in a symbiotic relationship with the host, and they are essential for maintaining oral health through producing antimicrobial substances and competing resources to inhibit pathogenic colonization (Jung and Lee, 2023). However, when the healthy microecological balance is disrupted, pathogenic germs proliferate freely, thereby triggering and worsening inflammation and tissue damage (Chen et al., 2022b; Gasmi Benahmed et al., 2022) (**Figure 1**) (**Table 1**). In diseased periodontal pockets, bacteria such as *Treponema*, *Porphyromonas*, *Fusobacterium* and *Prevotella* are highly abundant, and the number of bacteria such as *Fusobacterium* and *Selenomonas* increases with the periodontal pockets deepened (Gonçalves et al., 2012).

**Table 1:** Periodontitis and health-associated species ranked by importance.

Oral bacteria	Abundance changes	Characteristic	Reference
*Streptococcus sanguinis*	↓	A commensal bacterium	(Zhu et al., 2018)
*Actinomyces naeslundii*	↓	Widely exist	(Socransky et al., 1998)
*Gemella haemolysans*	↓	Inhibits the growth of *P. gingivalis*	(Miyoshi et al., 2021)
*Rothia aeria*	↓	Associated with periodontal health	(Khocht et al., 2021)
*Granulicatella adiacens*	↓	Widely exist	(Chen et al., 2022b)
*Rothia dentocariosa*	↓	Opportunistic pathogens of periodontitis	(Kataoka et al., 2014)
*Streptococcus mitis*	↓	Opportunistic pathogens of bacteremia	(Socransky et al., 1998)
*Actinomyces*	↓		(Chen et al., 2022b)
*Selenomonas*	↑	Dominant bacteria in deep periodontal pockets	(Gonçalves et al., 2012)
*Veillonella parvula*	↓		(Socransky et al., 1998)
*Corynebacterium durum*	↓	A commensal bacterium	(Chen et al., 2022b)
*Prevotella loescheii*	↓	Prevotella species in elevated levels in periodontitis	(Albaghdadi et al., 2021)
*Acinetobacter baumannii*	↓	A nosocomial, opportunistic pathogen that causes including meningitis, septicemia, endocarditis, and pneumonia	(Richards et al., 2015)
*Corynebacterium matruchotii*	↓	Regulating genome stability to anticancer	(Shen et al., 2022)
*Streptococcus infantis*	↓	Widely exist	(Yu et al., 2019)
*Fretibacterium*	↑	Can be used as a saliva-based diagnostic bacterial biomarker for periodontitis screening	(Khemwong et al., 2019)
*Treponema lecithinolyticum*	↑	Associated with rapidly progressive periodontitis	(Park et al., 2002)
*Bacteroidetes*	↑	Increased abundance in periodontal pockets	(Chen et al., 2022b)
*Peptostreptococcaceae*	↑	Located in the deep periodontal pocket	(Wei et al., 2019)
*Anaerolineae*	↑	Difficult to cultivate *in vitro*	(Chen et al., 2022b)
*Porphyromonas gingivalis*	↑	The most invasive pathogens associated with periodontitis	(Gasmi Benahmed et al., 2022)
*Streptococcus constellatus*	↑	Difficult to eradicate in periodontal pockets	(Socransky et al., 1998)
*Lachnospiraceae*	↑	Associated with oral-gut axis	(Cheng et al., 2024)
*Treponema*	↑	Important pathogenic bacteria of periodontitis	(Chen et al., 2022b)
*Tannerella forsythia*	↑	Oral pathogen strongly associated with periodontitis	(Ruscitto and Sharma, 2018)
Fretibacterium fastidiosum	↑	Located in the deep periodontal pocket	(Pérez-Chaparro et al., 2018)

Smoking is one of the most significant risk factors associated with the onset and progression of periodontal tissue inflammation. It impairs neutrophil chemotaxis and phagocytic function, disrupts humoral immunity, alters the expression levels of serum Immunoglobulin (Ig) G and IgE, increases the production of reactive oxygen species (ROS) and the release of proteases like collagenase and elastase, which directly contribute to periodontal tissue destruction (Huang and Shi, 2019). Research showed that smoking-induced subgingival dysbiosis occurs before clinical symptoms of periodontal disease (Tamashiro et al., 2023). Chemicals in tobacco such as nicotine and benzo[a]pyrene exhibit direct antimicrobial effects upon contact with microbial communities, leading to non-specific bactericidal effects that reduce normal microbiota and immune cell populations, creating opportunities for pathogenic bacteria to proliferate (McEachern et al., 2015). Furthermore, tobacco smoke and its chemicals significantly affect oxygen levels of local tissue and immunoglobulin concentrations of salivary (Tarbiah et al., 2019; Rinaldi et al., 2023), indirectly promoting changes in the microbial environment by altering habitats or disrupting host immunity and the biofilm formation process. Reduced immune function including diminished activity of oral neutrophils and macrophages, coupled with systemic chronic inflammation impairs the body’s microbial clearance, facilitating the colonization and growth of pathogenic bacteria. Smoking cessation remains the most effective method for prevention and treatment; however, the chronic inflammation caused by microbial imbalance is often difficult to reverse and may even be irreversible (Domagala-Kulawik, 2008).

Local irritants play a crucial role in shaping the microbiota within periodontal tissues. Despite significant progress in dental restorative materials and prosthetics over the past few decades, restoration failure rates continue to pose a challenge for both clinicians and researchers. Alterations in surface integrity, ion release and dissolution from dental fillings may promote bacterial adhesion, resulting in rougher restoration surfaces that influence biofilm formation and development (Yoshihara et al., 2017). Compared to smooth surfaces, rough surfaces provide more irregular areas, many of which are anaerobic regions and create an ideal environment for microbial colonization, facilitating the formation and maturation of oral plaque (Di Stefano et al., 2022). Additionally, deeper and larger depressions on rough surfaces increase the contact area, providing a more favorable interface for bacterial colonization and biofilm formation. These areas protect germs from shear forces, such as rinsing and brushing, during the initial reversible attachment phase, allowing for stronger and irreversible adhesion (Song et al., 2015). As a result, microbial colonies on rough restorative surfaces are more difficult to eliminate, leading to the formation of mature biofilms. On the other hand, dental restorative materials in the oral cavity are vulnerable to bacterial corrosion. Plaque can degrade the components of these materials, potentially compromising marginal integrity, promoting the development and progression of secondary caries, periodontitis and peri-implantitis (Li et al., 2014). The chemical properties of restorative materials can also affect microbial behavior. For instance, fluoride released from glass ionomer cement (GIC) reduces acid production, inhibits acid resistance and diminishes extracellular polymeric substance (EPS) formation in the plaque, particularly in cariogenic biofilms such as those formed by *Streptococcus mutans.* Fluoride also inhibits bacterial exopolysaccharide formation, preventing the increase of cariogenic bacteria, thereby suppressing the formation of cariogenic-dominant biofilms (Jung et al., 2016). Some polymeric components in composite resins have demonstrated inhibitory effects on biofilm formation (Ionescu et al., 2012); however, shortening the light-curing time could significantly increase the number of unpolymerized monomers on the material’s surface, which may contribute to increased colonization of *S. mutans* on composite resin surfaces (Brambilla et al., 2009).

Various stimuli ultimately result in the dominance of pathogenic bacteria. *Porphyromonas gingivalis*, one of the primary pathogens responsible for periodontitis, is known as a “keystone pathogen” or “ecopathogen”, signifying its critical role in initiating microbial dysbiosis during the disease’s progression (Gasmi Benahmed et al., 2022). Through the complex pathogenic mechanisms, *P. gingivalis* disrupts the balance of the entire microbial community and leads to the synergistic growth of other pathogens, primarily *Tannerella forsythia* and *Spirochetes*, which further intensify inflammation and tissue destruction (Mysak et al., 2014). Unlike other Gram-negative bacteria, the lipopolysaccharides (LPS) from *P. gingivalis* has a unique structure that activates Toll-like receptors (TLRs) *in vivo* (Jia et al., 2019), particularly TLR2 and TLR4. The activated TLR2 interacts with the capsular polysaccharide on the surface of *P. gingivalis*, resulting in bidirectional regulation of the immune response. This interaction combined with fimbriae and outer membrane vesicles enables *P. gingivalis* to evade host immune surveillance while inducing a fierce inflammatory response (Bostanci and Belibasakis, 2012; Lunar Silva and Cascales, 2021; Reyes, 2021; Zheng et al., 2021). Moreover, gingipains produced by *P. gingivalis* excessively activate the secretion of pro-inflammatory cytokines—primarily IL-1β, IL-6 and tumor necrosis factor-α (TNF-α). These chymotrypsin-like proteases, including Arginine-specific Gingipain and Lysine-specific Gingipain, also degrade defense proteins of parasitifer, such as immunoglobulins and complement, inhibiting the local immune response and facilitating periodontal tissue destruction (Bengtsson et al., 2015; Kadowaki, 2021). *P. gingivalis* is not confined to local effects in the oral cavity, it enters systemic circulation via bloodstream and contributes to the development and progression of systemic diseases by inducing systemic inflammation and abnormal immune activation (Klarström Engström et al., 2015).

*T. forsythia* forms the “Red Complex” in collaborates with *Treponema denticola* and *P. gingivalis*, colonizing and thriving in the anaerobic environment of periodontal pockets, and mutually contributing to the destruction of periodontal tissues (Suzuki et al., 2013). The Red Complex establishes a structured multispecies biofilm, which plays a key role in the progression of chronic periodontitis. This biofilm not only protects the bacteria from systemic immune responses but also enhances their resistance to antimicrobial treatments (Ong et al., 2017). Additionally, *T. forsythia* exacerbates inflammation through protease secretion and immune evasion. LPS on the cell wall of *T. forsythia* directly stimulates immune cells and lead to the secretion of pro-inflammatory cytokines such as IL-1, IL-6 and TNF-α (Pani et al., 2021). During active periods of periodontitis, the interaction between *T. forsythia* and *P. gingivalis* becomes more frequent, amplifying the inflammatory response. Studies indicated that all the rabbits co-infected with *P. gingivalis* and *T. forsythia* developed abscesses, while rabbits infected with either bacterium alone did not develop abscesses. Co-culture experiments results showed that *T. forsythia* exhibited a faster growth rate, suggesting a symbiotic nutritional relationship between the bacterial species (Verma et al., 2010).

*Aggregatibacter actinomycetemcomitans* is a Gram-negative facultative anaerobic bacillus, which is primarily found in the late stages of periodontitis or localized aggressive periodontitis (LAP) (Gholizadeh et al., 2017). This occurrence may be associated with early microbial colonization influenced by genetic factors (Åberg et al., 2015), and it is also considered a risk marker for the progression of periodontal attachment loss (Fine et al., 2007). The fimbriae and various adhesins produced by *A. actinomycetemcomitans* have been shown to be important for its proliferation in different ecological niches within the human oral cavity (Fine et al., 2010). After gaining the competitive advantage, *A. actinomycetemcomitans* primarily releases the leukotoxin LtxA to disrupt the anti-inflammatory function of leukocytes. The variability in virulence among different strains of this bacterium is related to gene expression, indicating that LtxA expression is regulated by both environmental and genetic factors (Johansson, 2011). Currently there are seven serotypes of *A. actinomycetemcomitans* (a–g) that have been identified based on the immunodominant antigen, which is an O-polysaccharide of the LPS. The JP2 genotype of *A. actinomycetemcomitans* harbors a 530-bp deletion in the promoter region of the leukotoxin operon, which is highly leukotoxic and has stronger pathogenic potential (Åberg et al., 2015). Macrophages are one of the primary producers of pro-inflammatory cytokines such as IL-1 and TNF. Studies have demonstrated that monocytes and macrophages produce pro-inflammatory cytokines, including TNF-α and IL-1β, in response to metabolic products of *A. actinomycetemcomitans* (Henderson et al., 2010). Macrophages exhibit heightened sensitivity to the pro-inflammatory response induced by LtxA, which specifically activates caspase-1, resulting in excessive secretion of IL-1β (Kelk et al., 2003, 2005); moreover, increased expression of inflammasome components could also be detected in co-cultures with *A. actinomycetemcomitans* (Belibasakis and Johansson, 2012). What’s more, LPS present in various pathogenic bacteria enhance pro-IL-1β expression in macrophages, which, along with inflammasomes and caspase-1, induces secondary stimulation of macrophages, leading to more IL-1β secretion and promoting periodontal tissue loss (Kelk et al., 2005, 2011). In contrast, significantly lower levels of IL-1 are often detected in the GCF of patients during the recovery period of periodontal treatment (Holmlund et al., 2004). The diversity of LtxA expression suggests that this toxin plays a significant role in the pathogenesis of aggressive periodontitis. Compared with patients with chronic periodontitis, adolescents (average age of 15 years) harboring highly leukotoxic strains of *A. actinomycetemcomitans* (both JP2 and non-JP2 genotypes) are at higher risk for localized periodontal attachment loss.

The ultimate consequence of uncontrolled periodontal tissue infection is alveolar bone loss. The primary objective of periodontal therapy is to prevent further disease progression, alleviate symptoms, reduce the risk of tooth loss, and explore the possibility of regenerating damaged periodontal tissue. In terms of etiological treatment, controlling and eliminating plaque-retentive factors is essential for reducing deep pocket inflammation and improving clinical attachment levels (CAL). In addition to full-oral mechanical debridement, the use of adjunctive chemical agents in combination with local and systemic antimicrobials is required to eliminate deep biofilms effectively in the subgingival area (Sanz et al., 2020). However, inappropriate medicines and treatment with chemical reagents can lead to oral microbiome dysbiosis. For example, chlorhexidine-containing medications may further reduce the pH of the acidic oral environment in periodontitis patients, which thus more suitable for treating gingivitis (Bescos et al., 2020). Beyond periodontal infections, dysbiosis can also cause oral mucosal ulcers, erosions and sensory abnormalities, while excessive use of antimicrobials may result in salivary glands inflammation, nerve numbness and allergic reactions (Herrera et al., 2002). Therefore, while eliminating the pathogenic bacteria, clinicians must consider systemic side effects, microbiological adverse reactions, and the significant risk of developing or exacerbating antibiotic resistance—all of which pose challenges for future periodontal therapy.

### 3.3 Alterations of the oral microbiota in peri-implant inflammation

Dental implants achieve long-term stability primarily through osseointegration. It represents a revolutionary innovation in the field of dentistry, aimed at replacing missing teeth and restoring masticatory, occlusal and aesthetic functions. Compared with traditional restorative methods, implant-based rehabilitation is considered a safer and more effective treatment option. The success rate of implant restoration is directly associated with advancements in implant surface process, design, materials and techniques (Alves et al., 2022). However, the incidence of implant-related complications has been increasing yearly, and the loss of hard and soft tissues around implants is influenced by multiple factors. For example, patients with a history of chronic or aggressive periodontitis are at a higher risk of implant failure (Sgolastra et al., 2015), while genetic polymorphisms may also increase the likelihood of developing peri-implantitis (Cardoso et al., 2024); additionally, poor oral hygiene is considered one more potential contributing factor to peri-implantitis (Ball and Darby, 2022), and smoking can also cause peri-implantitis as well as periodontitis.

The microbial composition of healthy peri-implant and periodontal sites overlap, but the proportions differ. The compositions of the biofilm around dental implants are similar to that surrounding adjacent natural teeth, making the teeth’s microbiome a “reservoir” for peri-implant biofilm (Belibasakis, 2014). However, there are also notable differences. Due to the direct integration of the implant with the alveolar bone, biofilms consisting of fewer species on healthy implants tend to have lower density and a simpler structure. These biofilms predominantly contain Gram-positive facultative anaerobic cocci and rods, also including *Actinomyces*, *Veillonella*, as well as low levels of *Clostridia* and *Bacteroides* (Hultin et al., 2002; Canullo et al., 2015; Jung and Lee, 2023). Studies have shown that healthy peri-implant tissues are primarily colonized by bacteria from the phyla *Firmicutes* and *Proteobacteria*. The *Streptococcus* and *Neisseria* species are particularly abundant, accounting for over 40% of the microbiota. Additionally, *Staphylococcus*, *Candida* and *Veillonella* are present in lower proportions in conditions simulating implant health (Sousa et al., 2022). This situation stands in stark contrast to the biofilms in peri-implantitis, which are denser, more diverse, and harbor more pathogenic species.

The risk factors and indicators for bone breakdown around implants and natural teeth share some similarities. Peri-implant infections can be categorized into peri-implant mucositis and peri-implantitis. Peri-implant mucositis is diagnosed if bleeding on probing (BOP) or suppuration is detected without radiographic evidence of alveolar bone loss exceeding initial remodeling. Similar to gingivitis around natural teeth, peri-implant mucositis shows a well-defined inflammatory infiltrate, primarily composed of vascular structures, plasma cells and lymphocytes located lateral to the junctional epithelium, without extending into the connective tissue of the alveolar crest (Araujo and Lindhe, 2018; Berglundh et al., 2018). If detected early and treated properly, peri-implant mucositis is regarded as a reversible condition. However, if not managed effectively, peri-implant mucositis can progress, causing bone resorption exceeding 2 mm from the initial remodeling and an increase in probing depth (PD) beyond 6 mm (Berglundh et al., 2018). At this stage, it is classified as peri-implantitis and the damage to surrounding tissues induces an inflammatory response mediated by the activation of innate immune cells such as macrophages, dendritic cells, mast cells and neutrophils. The release of pro-inflammatory cytokines like IL-1 and TNF-α is mainly promoted by neutrophils, extending the inflammation into the supporting bone, leading to both osteolytic and inflammatory tissue damage (Baseri et al., 2020), indicating that the inflammation has advanced to involve the surrounding alveolar bone.

Peri-implant infections are also regarded as polymicrobial diseases, with multispecies biofilms and their byproducts or metabolites as the primary drivers of the inflammatory response, commonly referred to as “peri-implantitis” (Lindhe and Meyle, 2008). Fundamentally, peri-implantitis is an endogenous mixed infection that occasionally involves atypical oral bacteria. This implies that external factors may also contribute to the infection, although defining the extent of this contribution remains difficult (Kumar, 2019). In long term follow-up studies, peri-implantitis was observed in 28-56% of implant recipients and different degrees of infections were detected in 12-43% of implant sites (Roos-Jansåker et al., 2006; Zitzmann and Berglundh, 2008). Among these cases, approximately 12.4% of peri-implant diseases were directly associated with dental plaque, 17.1% were related to surgical factors, 32.4% were linked to prosthetic factors and 13.3% were due to biomechanical factors, with about 25% involving a combination of these factors (Canullo et al., 2017). It is suggested that the causes of peri-implant diseases are complex, involving not only patient-specific conditions but also the risk of bacterial infection during surgery or prosthetic restoration. Research has shown that patients with a history of periodontitis are more susceptible to peri-implant diseases, with bacteria potentially migrating from the periodontal tissues of natural teeth to the surrounding implant area (Cho-Yan Lee et al., 2012; Pjetursson et al., 2012). The unique structure of implants causes inflammation to progress more rapidly. Oral microbiome dysbiosis accelerating the production of cytokines, chemokines, prostaglandins and proteases, further exacerbating tissue destruction and broadening the extent of the lesions (Komatsu et al., 2020; Giro et al., 2021). A research found that implants in the posterior region are six times more likely to develop inflammation than those in the anterior region, with the majority of periodontal pathogens, especially the red complex, being present around implants (Albertini et al., 2015). As inflammation progresses, it can lead to implant loss, maxillary sinusitis (Park et al., 2019), jaw fractures, and other complications including infections in adjacent implants or natural teeth (O’Sullivan et al., 2006). Furthermore, prolonged exposure of the implant’s metal surface to biofilm or physiological friction at the implant-abutment interface can cause corrosion and wear of the implant, releasing titanium ions, microparticles, and even nanoparticles that may affect surrounding tissues and enhance macrophage-mediated inflammation (Apaza-Bedoya et al., 2017; Pettersson et al., 2017). This microbial-induced biocorrosion of the implant surface could exacerbate titanium particle release and bio-implant neopathy (Mombelli et al., 2018), ultimately resulting in pathological bone resorption. Apatzidou et al. (Apatzidou et al., 2017) used 16S rRNA sequencing to analyze the microbial composition of peri-implantitis sites and non-adjacent healthy periodontal sites in patients undergoing periodontal treatment. Their results indicated that healthy periodontal sites had greater microbial diversity, associated with higher levels of *Actinomyces* and *Streptococcus*. In contrast, *Prevotella* and *Porphyromonas* were closely linked to the severity of peri-implantitis.

Studies have shown that in early-stage peri-implant mucositis, *Escherichia coli* still accounts for nearly 50% of the bacterial population in contrast to the microbiota around healthy implants, where the proportion of *Neisseria meningitidis* (HMT669) significantly decreases, and pathogenic bacteria such as *Porphyromonas* and *Treponema* remain at low levels (Sousa et al., 2022; Feng et al., 2024). While *Peptostreptococcus micros* is typically present in low abundance around healthy implants, it becomes enriched in peri-implantitis sites (Jung and Lee, 2023). Additionally, several less pathogenic bacterial species have been detected at higher levels in peri-implantitis, including *Alloprevotella* sp. (HMT473), *Enterobacteriaceae* spp., *Parvimonas micra* (HMT111), *Peptostreptococcus stomatis* (HMT112), *Gemella morbillorum* (HMT046), *Prevotella saccharolytica* (HMT781), *Pasteurellaceae* spp., *Parvimonas* spp., and *Klebsiella pneumoniae* (HMT731). The anaerobic bacterial around implants usually does not occur in the initial stages of inflammation but begin to increase after two weeks and reach higher levels by the third week. At the same time, the abundance of *Proteobacteria* decreases significantly (Sousa et al., 2022), suggesting that the progression has already evolve from mucositis to peri-implantitis, along with a significant change in bacterial composition. As the inflammation continues to progress, levels of *Leptotrichia*, *Propionibacterium* and *Prevotella* decrease, while *Actinomyces*, *Peptococcus*, *Campylobacter*, non-mutans *Streptococcus*, *Butyrivibrio* and *S. mutans* are found at elevated levels compared to healthy implants (Kumar et al., 2012). Another study found that *T. forsythia* was commonly detected among peri-implantitis patients, with 24% of patients testing positive for *Prevotella intermedia*, and 39% of patients testing positive for *T. denticola* of cases. Moreover, mixed bacterial infections were detected at inflamed peri-implant sites, such as *P. gingivalis* + *T. forsythia* (33%) and *T. forsythia* + *T. denticola* (24%). Notably, *A. actinomycetemcomitans* was not found in any peri-implantitis patients in this study (Albertini et al., 2015), suggesting that titanium implants may influence the colonization of periodontal pathogens and even change the microbial diversity of surrounding tissues. In addition to anaerobic periodontal bacteria, some rare oral microorganisms such as *Pseudomonas aeruginosa* and *Staphylococcus aureus*, exhibit higher adhesion to titanium surfaces and could even cause early postoperative infections likely due to the multidrug resistance of those bacteria (Truong et al., 2010; Cobo et al., 2011; D’Ovidio et al., 2011; Sendra et al., 2024). Interestingly, the use of certain low-concentration oral disinfectants in early postoperative open wounds can promote the growth of these bacteria, particularly *P. aeruginosa* (Morales-Fernández et al., 2014). Some fungi can also colonize the peri-implant pocket. This situation emphasizes the need for comprehensive antimicrobial strategies to protect the delicate microbial environment around implants. For instance, *Candida albicans* can adhere to and colonize mucosal tissues or inert materials, causing not only inflammatory damage to surrounding tissues but also forming complex biofilms with other pathogens. These biofilms worsen the persistence and severity of infections, making treatment more challenging (Salerno et al., 2011). Additionally, viruses such as human cytomegalovirus, human herpesvirus types 4 and 5, and Epstein–Barr virus type 1 as part of the biofilm complex have been identified in peri-implant inflammatory tissues. However, there is currently no definitive evidence proving their direct association with the etiology of inflammation (Pérez-Chaparro et al., 2016).

### 3.4 The correlation between oral microbiota and intestinal microbiota

Given the direct connection between the oral cavity and the gastrointestinal tract, the oral microbiota unavoidably interacts with and influences the gastrointestinal microbiota. But research on the influence of gut microbiota on the oral microbiota remains limited. The gut microbiota as the largest microbial community in the human body, is primarily composed of Firmicutes, Bacteroidetes, Actinobacteria and Proteobacteria. Firmicutes are Gram-positive bacteria with over 200 genera constituting 60%–80% of the community, the most important of which include *Ruminococcus*, *Clostridium*, *Eubacterium*, *Lactobacillus*, *Faecalibacterium*, *Roseburia* and *Mycoplasma*; Bacteroidetes are primarily Gram-negative including the genera *Bacteroides*, *Prevotella* and *Xylanibacter*, comprising 20%–30% of the microbiota; Actinobacteria are Gram-positive represent less than 10% and notably include *Bifidobacterium*; Proteobacteria are Gram-negative make up less than 1% of the microbiota and consist of genera such as *Escherichia*, *Desulfovibrio* and *Enterobacter*; the phylum Verrucomicrobia occupies an even smaller proportion of the gut microbiota, represented by genera like *Akkermansia*, *Fusobacterium* and *Cyanobacteria* (Bao et al., 2022).

**Figure 2:**
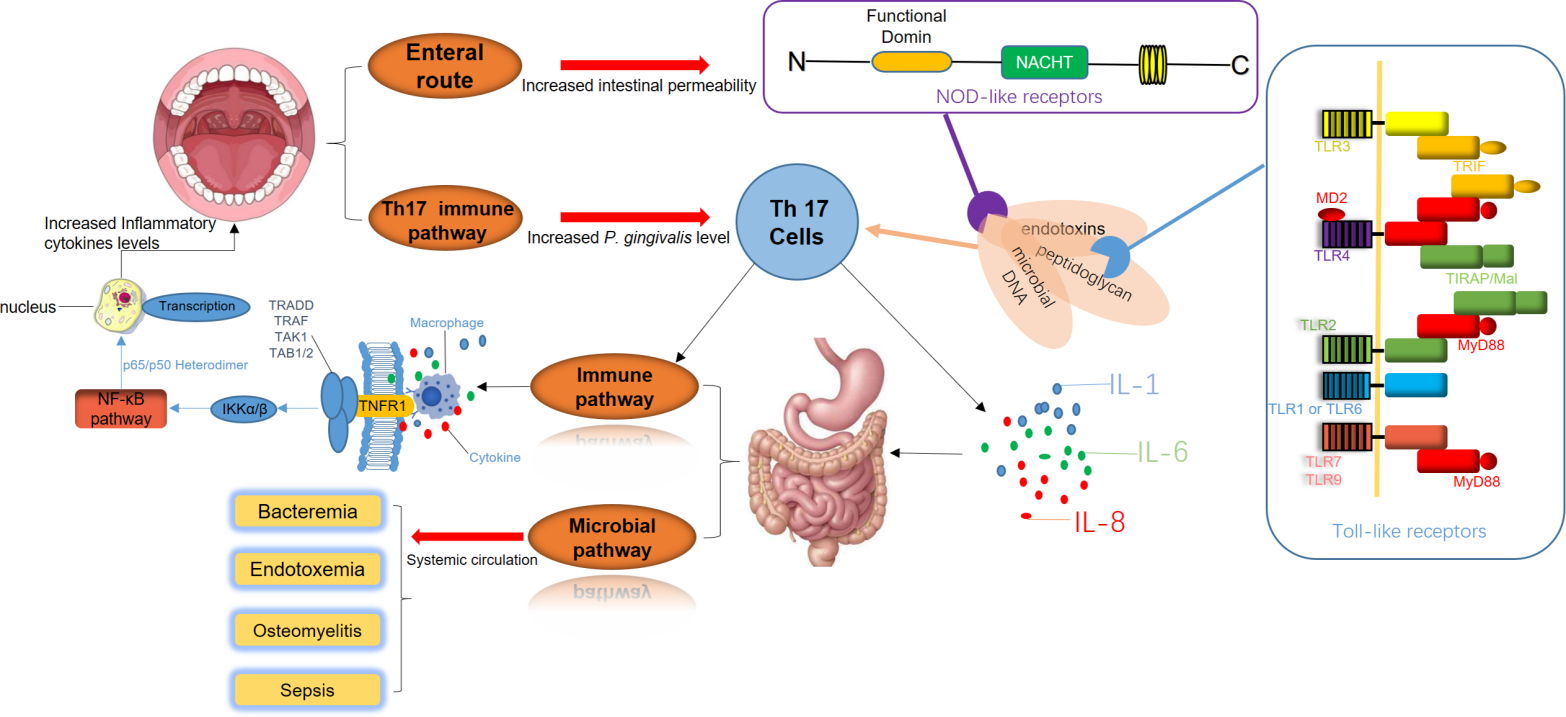
The disruption of periodontal and gastrointestinal microbiota is a significant trigger of systemic immune responses. Periodontal pathogens release virulence factors such as endotoxins, peptidoglycans, and microbial DNA, which directly enter the digestive system. These components interact with Toll-like receptors (TLRs) on the intestinal epithelial surface and Nod-like receptors (NLRs) within cells, initiating inflammatory signaling cascades. Simultaneously, Th17 cell-mediated immune pathways are activated, promoting the secretion of pro-inflammatory cytokines, including IL-1, IL-6, and IL-8. This inflammatory milieu intensifies intestinal inflammation, creating a feedback loop that further amplifies the activation of Th17 cells and macrophages. As a result, systemic immune pathways are enhanced, elevating inflammatory cytokine levels and accelerating the progression of periodontal inflammation. Additionally, bacteria and their inflammatory mediators from periodontal sites can enter the systemic circulation. This process leads to bacteremia, endotoxemia, and in severe cases, sepsis. These systemic infections can result in severe complications, including osteomyelitis and widespread bacterial dissemination.

According to a relevant report, the oral microbiota of mice in the early stage of diabetes resembles that of healthy mice. However, with rising blood glucose levels, the pathogenic potential of the oral microbiota increases, evidenced by significant alteration in β-diversity and a marked increase in periodontitis-related bacteria (Xiao et al., 2017). In addition, patients with inflammatory bowel disease (IBD) are frequently observed to concurrently suffer from chronic periodontitis, as both diseases involve inflammation closely related to host immune responses and microbial dysbiosis (Muhvić-Urek et al., 2016; de Mello-Neto et al., 2021). Studies indicate a higher detection rate of *Fusobacterium nucleatum* in the colons of IBD patients and the strains isolated from inflamed tissue shows greater invasiveness compared with those from healthy control tissue (Strauss et al., 2011). In addition, compared with control subjects, IBD patients have lower IL-4 expression levels in GCF and higher IL-18 levels in serum. Severe IBD is often associated with worse alveolar bone resorption, likely due to reduced IL-4 in GCF limiting the inhibition of osteoclast activity (Figueredo et al., 2017). Therefore, it can be inferred that cytokines resulting from gut dysbiosis are related to systemic inflammation and probably impact the development and progression of chronic periodontitis (**Figure 2**). On the other hand, the imbalance of oral microbiota also affects the microbial homeostasis throughout the body system. Under physiological conditions, stomach acid and bile generally inhibit the migration of oral microbiota to the gastrointestinal tract, but in patients with periodontitis or peri-implantitis, certain acid-resistant bacteria in the oral cavity could withstand H^+^ (the low pH) conditions and protease activity, potentially increasing the systemic spread of these pathogens (Liu et al., 2021). In animal models with periodontitis, genomic DNA of *F. nucleatum* has been detected in various systemic organs including the blood, heart, lungs and liver. Accordingly, some researchers have proposed the “oral-gut-liver axis” hypothesis, suggesting that periodontopathic microorganisms might infiltrate the systemic circulation via damaged gingival pocket epithelium or collagen fibers and matrix within adjacent connective tissue. This infiltration could lead to bacteremia or endotoxemia, enabling pathogenic bacteria to spread and colonization in various organs throughout the body (Acharya et al., 2017).

Apart from the effects of salivary flow and blood circulation pathways, dysbiosis of the oral microbiota could also directly influence immune pathways by disrupting the balance between innate and adaptive immunity, potentially leading to immune-mediated inflammatory diseases (IMIDs). T helper cells-17 (Th17 cells) and the IL-17/IL-23 signaling axis play critical roles in adaptive immune responses in this process. Moreover, microbial imbalance is considered to be a primary driver of protective immune responses in barrier tissues. The periodontal pathogens accumulate in the gut due to oral inflammation. Their toxic byproducts and pro-inflammatory cytokines, such as TNF-α and IL-1β produced by local tissues result in immune responses in the gut barrier, with IL-17 exerting a particularly notable effect (Kitamoto et al., 2020). Besides, elevated IL-17 expression in GCF have been observed in patients with periodontitis, suggesting that gut microbiota dysbiosis induces subsequent endotoxemiam, and the Th17 cell-mediated gut immune response may underlie the relation between chronic periodontitis and systemic diseases (Beura et al., 2016).

### 3.5 Regional immunity caused by periodontal homeostasis and imbalance

**Figure 3:**
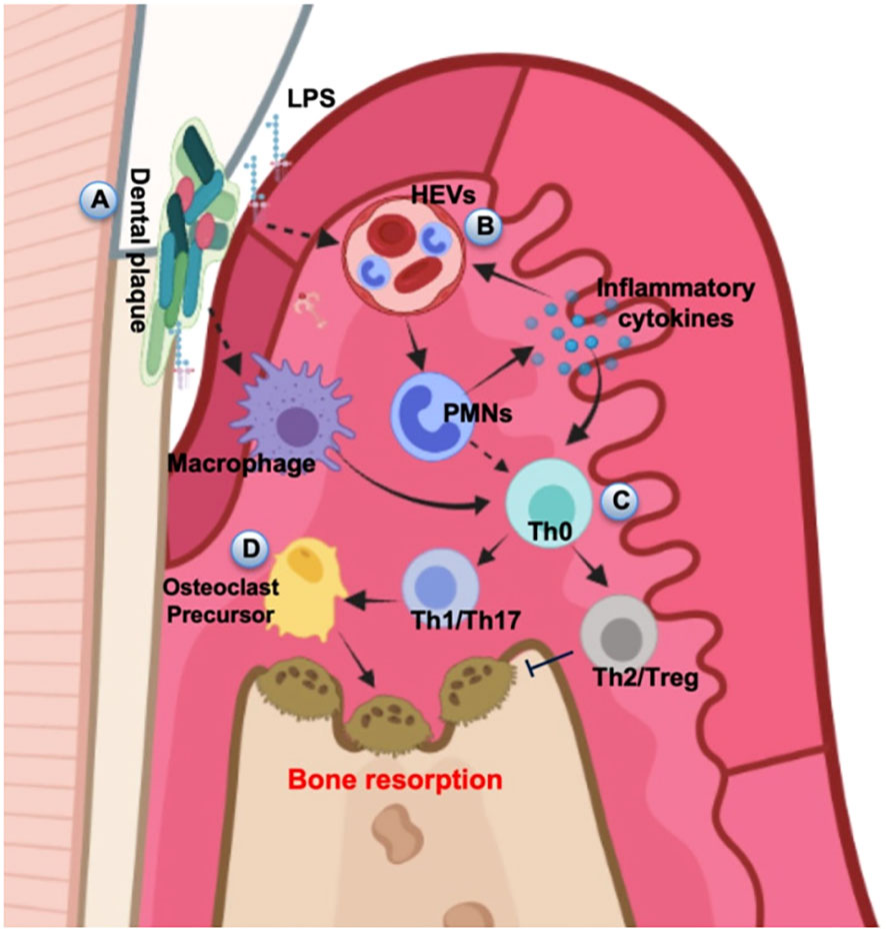
Schematic diagram of alveolar bone destruction in inflammatory response. **(A)** Initially, pathogenic microorganisms and toxic products in dental plaque stimulate periodontal tissue, directly inducing macrophages to cause local inflammation and immune response. **(B)** LPS and inflammatory cytokines activate the HEVs leading to vascular hyperpermeability and PMN transmigration, and this process can also provide feedback to regulate the release of inflammatory cytokines. **(C)** Upstream stimulation drives T helper cells to differentiate into several subsets for cellular immunity. **(D)** The amplification of local immune response leads to the development of inflammation and results in the progression of periodontal destruction and bone resorption. The diagram was devised from (Di Stefano et al., 2022).

The periodontal microbiota, epithelial barrier, soft tissue extracellular matrix (ECM) and bone-tissue coupling system form an intricate regulatory network that collectively sustains periodontal homeostasis. Since the concept of “homeostasis medicine” was proposed (Wang and Qin, 2022), research on the etiology, treatment, and preventive strategies for periodontal and peri-implant inflammation has altered from localized focus to the systemic approach. The body regulates periodontal environment stability via receptors on various immune cell surfaces, signaling pathways and secreted cytokines. In healthy physiological states, subgingival plaque exhibits low pathogenicity, while the physical barrier of periodontal mucosa remains intact. The moderate immune response primarily involves neutrophils, macrophages and T-cell subsets (γδT-cells) to execute effective surveillance, control local microbiota and tolerate symbiotic microbes and harmless antigens to a certain extent (Belkaid and Harrison, 2017). Periodontal supporting tissues also undergo physiological remodeling in response to moderate external stimuli without inducing significant tissue destruction. The dysregulation of periodontal homeostasis begins with the localized colonization of core periodontal pathogens, which destabilizes the epithelial homeostasis. Pathogens disrupt regional immune surveillance and microbial control mechanisms, heightening the pathogenicity of the subgingival microbiota and disturbing the microbiome-epithelium balance. Stimulated by microbial activity, tissue and resident immune cells release substantial levels of pro-inflammatory mediators such as cytokines and chemokines, which subsequently activate regional immune responses through positive feedback (Hajishengallis, 2015). In this phase, cytokines produced by the residential cell population have the main function to stimulate cells migration to sites of infection and enhance the expression of adhesion molecules for neutrophils on the internal vessel surfaces and increase the synthesis of other proinflammatory cytokines (Di Stefano et al., 2022). Besides, the expression of ECM-metabolizing enzymes is upregulated in this state, while ECM synthesis is impaired, ultimately compromising gingival soft tissue homeostasis; the osteoblast-osteoclast coupling balance is disrupted, resulting in osteoclast activation and subsequent alveolar bone resorption (**Figure 3**).

Both dental implants and natural teeth breach the periodontal mucosal barrier and expose within the oral microbial environment. However, this does not mean that this barrier cannot play a practical role. The gingival sulcular epithelium responds to subgingival microbial stimuli by releasing defensins, antimicrobial peptides and cytokines, which activate regional immunity to manage microbial communities and support periodontal homeostasis (Ross and Herzberg, 2016). The junctional epithelium at the sulcus base has a weaker barrier function. Neutrophils and tissue fluid containing antimicrobial agents and cytokines permeate into the gingival sulcus, forming GCF, which further contributes to periodontal stability (Moutsopoulos and Konkel, 2018); however, around implants, the lack of robust junctional epithelium enhances susceptibility to severe inflammatory assaults. Furthermore, solitary chemosensory cells dispersed within the epithelial layer could detect microbial stimuli via bitter taste receptors, thereby performing immune surveillance functions (Zheng et al., 2019). Neutrophils are the predominant innate immune cells in healthy gingiva, where both a deficiency and overactivity of neutrophils can result in periodontal damage (Xiao et al., 2017; Hajishengallis, 2020). When gingival sulcus and junctional epithelial cells are stimulated by pathogenic bacteria, they induce specific immune cells to express pro-inflammatory mediators, activating downstream signaling pathways that drive extensive neutrophil infiltration. This neutrophil-driven pathway of tissue damage mediated by chemotactic factors is termed the “matrix-neutrophil axis” (Hajishengallis and Chavakis, 2013; Williams et al., 2021). Moreover, neutrophils in the gingiva of inflammatory patients remain hyperactivated and highly reactive that is difficult to reverse in a short term following periodontal therapy (Ling et al., 2015). The above observations indicated that epithelial cells, fibroblasts and other tissue cells not only experience inflammatory damage but may play the role of initiators or signal transmitters during the progression of homeostatic imbalance, and the maintenance of periodontal homeostasis may necessitate a balanced level of neutrophil infiltration. During adaptive immunity, Th17 cells serve as the primary source of IL-17 at mucosal barriers and locally destabilized periodontal tissues are often accompanied by a marked increase in IL-17 expression and Th17 cell infiltration (Beklen et al., 2007; Kitamoto et al., 2020). IL-17 enhances neutrophil adhesion to vascular endothelium, promotes fibroblast secretion of ECM degrading enzymes, and upregulates RANKL expression to activate osteoclasts, thereby disrupting soft tissue metabolism and bone coupling homeostasis (Kotake et al., 2012; Miossec and Kolls, 2012); elevated local immune activation induces substantial expression of pro-inflammatory cytokines, including IL-1 and IL-17, which in turn activate osteoclasts and inhibit osteoblast function through the RANKL/NF-κB/osteoprotegerin signaling axis, causing bone resorption in periodontitis (Lin et al., 2014; Pan et al., 2019). Besides, studies reported that IL-17 and IL-21 secreted by Th17 cells could induce differentiation, B-cell proliferation and IgG production (Ettinger et al., 2007; Hickman-Brecks et al., 2011). Neutralization of IL-17 antibodies has been shown to alleviate alveolar bone resorption caused by periodontitis (Hajishengallis, 2020). Thus, Th17 cells are widely recognized as critical contributors to immune homeostasis imbalance in the periodontal region, mediating periodontal instability through multiple mechanisms. Another inflammatory mediator, IL-18, activates the NF-κB pathway, leading fibroblasts, neutrophils and monocytes to release matrix metalloproteinase (MMPs), which result in periodontal tissue degradation (Beklen et al., 2007; Wang et al., 2019). In addition, osteoblasts and osteoclasts work in tandem to establish bone-coupling homeostasis, ensuring continuous bone formation and remodeling. Notably, approximately 50% of T cells and 90% of B cells in the gingival tissues of periodontitis patients express RANKL, implying that lymphocyte-derived immune cells may be a significant source of RANKL, contributing to bone-coupling imbalance (Kawai et al., 2006). Through disruption of bone-coupling homeostasis, B cells may actively drive periodontal instability.

The periodontal barrier encounters diverse stimuli such as mechanical forces from mastication and food antigens. The composition of local subgingival microbiota is highly complex, but the barrier function is relatively weak. The body needs to maintain local homeostasis through active and complex regulatory mechanisms. Based on the previously discussed factors leading to the maintenance and disruption of periodontal homeostasis, a range of novel strategies aimed at restoring periodontal stability is currently under investigation. These strategies include controlling pathogenic microbiota, modulating immune responses in the periodontal region and guiding the regeneration of soft and hard tissues. Among these, suppressing the progression of immune-mediated inflammation driven by pathogenic microorganisms remains the primary focus.

### 3.6 The differences and similarities between periodontitis and peri implantitis

**Figure 4:**
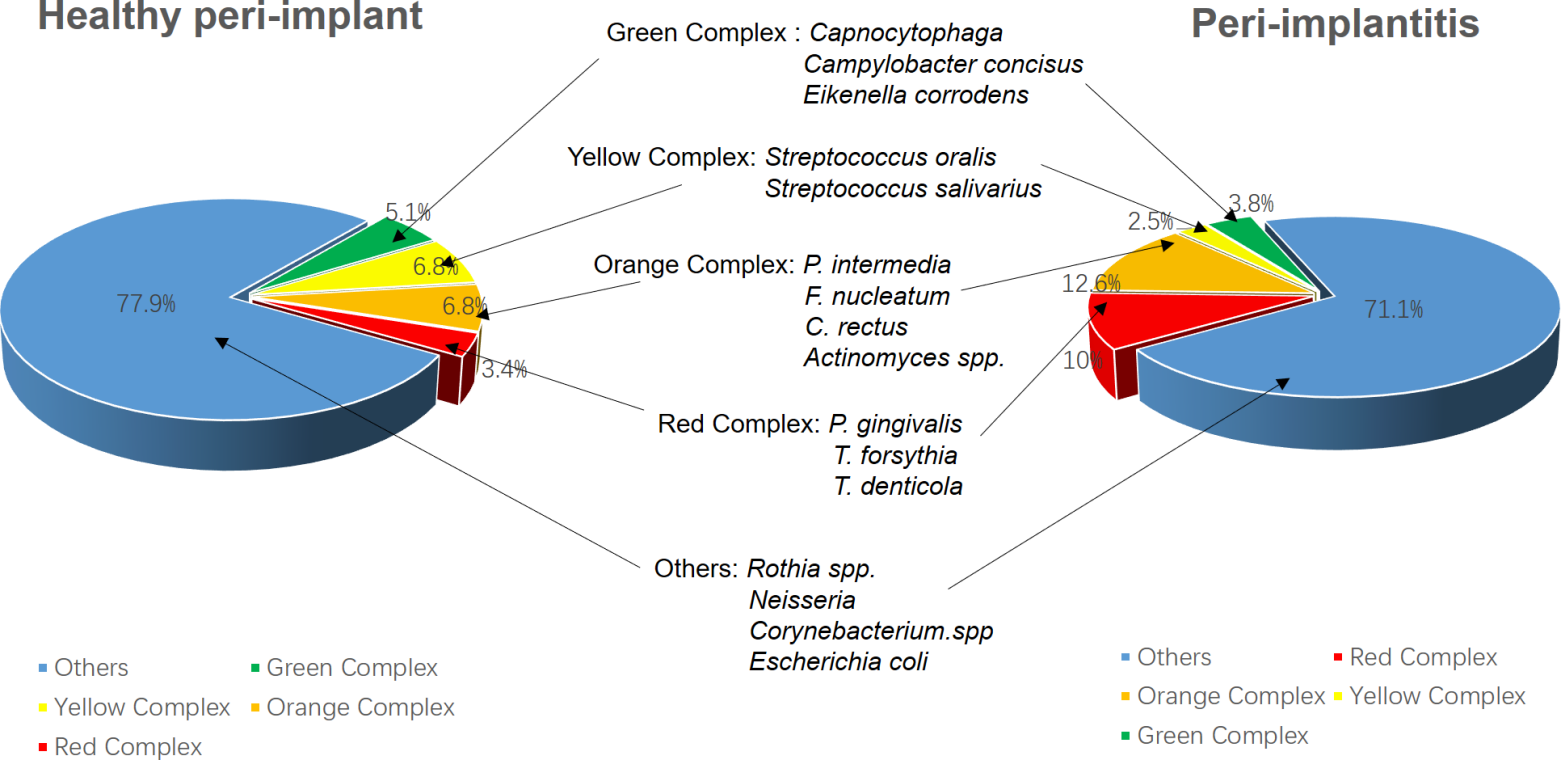
The diversity of microbial communities shows no significant differences between peri-implantitis patients and those with healthy peri-implant tissues. However, peri-implantitis is characterized by a notable increase in pathogenic bacterial abundance; meanwhile, the proportion of opportunistic pathogens from the yellow and green complexes decreases, suggesting that peri-implantitis is primarily dominated by the red complex. The data were derived from (Al-Ahmad et al., 2018).

The peri-implant tissues harbor approximately 400 microbial species, which is significantly fewer than those found in healthy periodontal tissues. However, research showed that the microbial characteristics of healthy peri-implant and periodontal sites share greater similarities compared to inflamed regions (Zheng et al., 2015). The distinctions from health to disease in the periodontal and peri-implant tissues are not determined solely by the presence or absence of specific pathogens but rather reflected in taxonomic changes across the entire microbial community. Furthermore, the changes in microbial diversity are marked by a reduction in the number of bacterial species under healthy conditions and a gradual increase in complexity as disease progresses, underscoring the importance of maintaining a balanced microbiome to prevent disease onset (Belibasakis and Manoil, 2021; Jung and Lee, 2023). The necessity of regular monitoring and early intervention has been proposed to protect the health of periodontal and peri-implant tissues. In the first few months following implant placement, the biofilm’s taxonomic composition around the implant shows only slight differences; however, microbial diversity is lower than that around adjacent teeth (Payne et al., 2017). Alpha diversity analysis reveals no significant differences in the diversity of the subgingival plaque community between patients with peri-implantitis and those with peri-implant health, suggesting that pathogenic bacteria associated with peri-implant inflammation may be present from the outset (Yu et al., 2019) (**Figure 4**). However, compared to healthy teeth, elevated levels of *Prevotella*, *Treponema*, *Leptotrichia*, *S. mutans*, *Butyrivibrio*, *Catonella*, *Propionibacter* and *Lactococcus* are observed in the peri-implant sulcus during the early phase of implantation (Kumar et al., 2012), suggesting an increased risk of inflammation. While non-specific opportunistic pathogens are less abundant around healthy tissues, *S. aureus*, *P. aeruginosa*, and *C. albicans* can still be detected. Additionally, bacterial diversity increases in inflamed peri-implant tissues with more opportunistic pathogens including *S. intermedius*, *S. aureus*, *S. mitis* and *Haemophilus influenzae* being identified (Padial-Molina et al., 2016). Furthermore, almost all cases of peri-implant inflammation were accompanied by bleeding on probing, and the probing results closely matched the average values recorded from periodontal tissues. A study found that when comparing pathogens between implant and tooth samples from the same individual, 54% of patients showed identical results in both sample types, while 45% showed differing results. In 39% of patients, the implant sample was positive for periodontal pathogens, while the tooth sample was negative. Only 6% of patients showed a positive tooth sample and a negative implant sample (Albertini et al., 2015).

**Figure 5:**
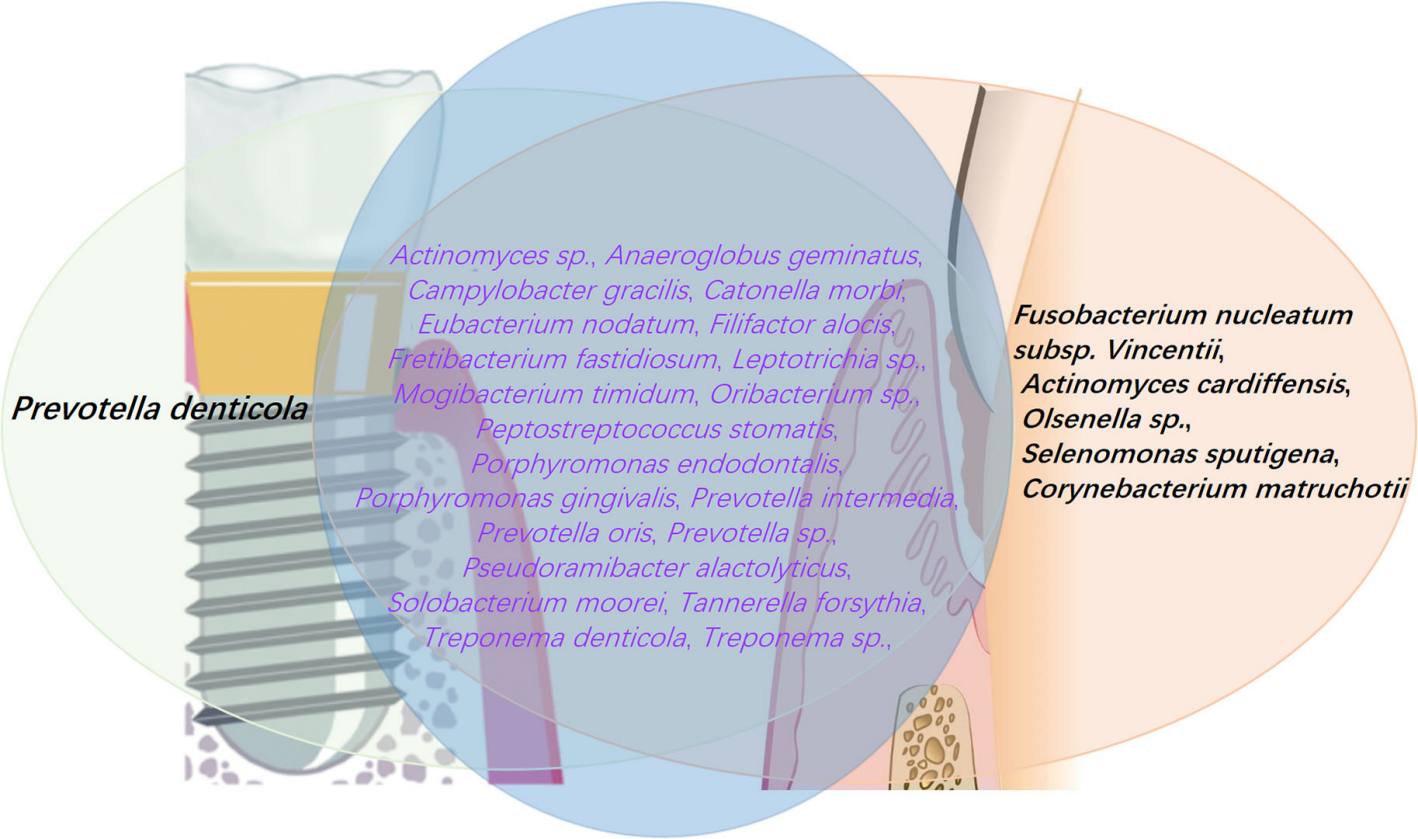
Periodontitis is characterized by the presence of 26 high-prevalence pathogens, 21 of which are also found in peri-implantitis. However, certain pathogens are unique to periodontitis, including *F. nucleatum subsp*. *Vincentii*, *A. cardiffensis*, *Olsenella* sp., *S.* sp*utigena*, and *C. matruchotii*. Conversely, *P. denticola* is predominantly associated with peri-implantitis, playing a key role in the inflammatory processes and biofilm development characteristic of this condition. The data were derived from (Komatsu et al., 2020).

The immunological events driving peri-implant infections are qualitatively similar with those in periodontal infections, primarily involving Gram-negative bacteria. However, peri-implant infections have a broader inflammatory scope and lead to more rapid tissue destruction (Belibasakis et al., 2015). Natural teeth are anchored to the alveolar bone by the periodontal ligament (PDL), while dental implants are directly anchored to the alveolar bone through osseointegration. The lack of the PDL around implant limits the blood supply, reducing the availability of nutrients and immune cells necessary to combat early bacterial infections (Araujo and Lindhe, 2018; Berglundh et al., 2018). Additionally, the fibers in the alveolar ridge connective tissue are arranged circumferentially rather than vertically, impairing the capacity of the physical barrier against bacterial invasion into the submucosa and leaving the peri-implant tissues in a more fragile “open wound” state. Subtle variations across multiple factors result in slight differences between the microbial communities of peri-implantitis and periodontitis. In general, several critical periodontal pathogens, including *P. gingivalis*, *T. denticola*, *T. forsythia*, *A. actinomycetemcomitans*, *P. intermedia*, *F. nucleatum*, and *Campylobacter* spp., are strongly associated with peri-implantitis (Zheng et al., 2015). Despite significant overlap, the microbiota of peri-implantitis exhibits distinct differences compared to that of periodontitis. Research has identified approximately 22 high-abundance pathogenic species in peri-implantitis, 21 of which are also commonly found in periodontitis. However, certain species that are prevalent in periodontitis, such as *F. nucleatum subsp*. *vincentii*, *A. cardiffensis*, *Olsenella* spp., *Selenomonas* sp*utigena*, and *Corynebacterium matruchotii*, are rarely detected in peri-implantitis (Komatsu et al., 2020) (**Figure 5**). Smoking profoundly affects the oral microbiome, altering its composition in tissues. In inflammation of both peri-implant and natural periodontal, *P. gingivalis* and *T. denticola* are more prevalent in smokers than in non-smokers, both the two types of pathogens with prevalence rates of 90% and 60% in smokers compared with those 69% and 34% in non-smokers, respectively; however, *T. forsythia* and *P. intermedia* were more frequently observed in non-smokers, with prevalence rates of 69% and 47%, respectively, compared with 60% and 30% in smokers; on the other hand, for some opportunistic microorganisms, *P. aeruginosa* is more commonly found in smokers than in non-smokers, with the prevalence rates of 20% and 9%, respectively (Albertini et al., 2015). Although both periodontitis and peri-implantitis are plaque-induced inflammatory conditions, some other local factors may also be associated with this complication due to their role in promoting plaque retention. These modifiable factors including surgical factors (e.g., improper implant placement, failure of bone reconstruction), prosthetic factors (e.g., inadequate design of implant-supported restorations, improper distribution of stress in the restoration), or biomechanical factors (e.g., overload, excessive occlusal pressure) may contribute to the development of unfavorable conditions, promoting the transition from physiological bone loss to peri-implant disease. Therefore, disease classification has to integrate factors that related to surgery, prosthetics and biomechanics to better address these issues (Canullo et al., 2017).

### 3.7 The impact of non-mechanical treatment of inflammation on microorganisms

Mechanical debridement (MD) alone often insufficient to meet both efficiency and minimally invasive standards required for treating peri-implant diseases. Combining non-surgical treatments with MD for better outcomes has become an increasingly common approach (Dentino et al., 2013; Majzoub et al., 2021). However, different non-surgical options exhibit varying levels of efficacy, making it necessary to explore the optimal treatment combinations and understand the differences in therapeutic outcomes to guide clinical practice. Research have shown that all isolated strains of *P. aeruginosa* are sensitive to ciprofloxacin, ceftazidime, and aminoglycosides, while the isolated strains of *S. aureus* are sensitive to methicillin. Nevertheless, antibiotic resistance remains an unavoidable problem, and the non-selective bactericidal effects of antibiotics are directly linked to microbial dysbiosis (Alves et al., 2022). Probiotic supplements administered as an adjunct to supragingival scaling and root planing have been shown to partially suppress the proliferation of pathogenic bacteria, thereby mitigating microbial dysbiosis (Vivekananda et al., 2010; Alves et al., 2022). As such, Probiotics are regarded as a safe and effective biological intervention, with studies in animal models certified that specific probiotic strains administered orally effectively inhibit alveolar bone loss. These probiotics influence the overall development and stability of the microbiota in the oral cavity by modulating the colonization potential of pathogens, thereby supporting and stabilizing the host immune system and reducing the production of pro-inflammatory cytokines (Lauritano et al., 2019; Yang et al., 2020a). Although these bacteria may not directly eradicate pathogens, this approach may provide some clinical efficacy in reducing periodontal pocket depth in periodontitis (Peña et al., 2019). Research indicated that *Rothia* species harbor nitrate reductase enzymes, which catalyze the reduction of nitrate (NO_3_^−^) to nitrite (NO_2_^−^). In this process, *Rothia* directly utilize nitrate-rich foods as an additional energy source, a capability particularly prominent in the hypoxic or anoxic conditions of the gingival sulcus. The nitrite is subsequently metabolized into nitric oxide (NO), a bioactive molecule with potent antimicrobial properties, which reduces the pathogenic bacterial burden and mitigates periodontal inflammation (Mazurel et al., 2023). Furthermore, nitrate-metabolizing bacteria contribute to pH regulation and redox balance in the gingival sulcus, improving the microbial habitat conditions and supporting the co-growth of other beneficial bacterial species (Yamamoto et al., 2024). The lactic acid bacterium *Lactobacillus reuteri* (primarily Gram-positive) has been demonstrated to outcompete pathogens for adhesion to epithelial tissues and gain a colonization advantage. It reduces soft tissue damage by inhibiting the production of MMP-8 in periodontal tissues (İnce et al., 2015), induces controlled oxidative stress in surrounding cells through 3-hydroxypropionaldehyde formation to protect native tissues (Tekce et al., 2015), and decreases the expression levels of pro-inflammatory cytokines such as TNF-α, IL-1β, IL-6 and IL-8 in patients with peri-implant mucositis, effectively inhibiting the growth of *A. actinomycetemcomitans*, *P. gingivalis*, and *P. intermedia* (Kõll-Klais et al., 2005; Szkaradkiewicz et al., 2014), Moreover, *P. gingivalis*, *P. intermedia*, *Streptococcus salivarius*, and *S. aureus*, among other common pathogens associated with periodontitis or peri-implantitis are highly sensitive to these lactic acid bacteria (Mulla et al., 2021). The most common oral bacterium, *S. salivarius*, has been shown to have beneficial effects on implant-associated biofilms. Probiotics derived from *S. salivarius* produce bacteriocins that inhibit quorum sensing signals and reduce *S. intermedius* biofilm formation on titanium implant surfaces (Vacca et al., 2020). Although *Streptococcus sanguinis* and *Streptococcus uberis* are relatively low in abundance, they could produce hydrogen peroxide to change the local environment, effectively inhibiting the growth of *A. actinomycetemcomitans* (Laleman et al., 2015). To sum up, these probiotics may be considered adjuncts in non-surgical treatments to prevent biofilm-related oral diseases. Currently, no specific studies revealed whether interactions exist between probiotics and fungi (Martorano-Fernandes et al., 2020).

Due to the rough and helical structure of implant surfaces, ultrasonic scaling is often insufficient for complete bacterial removal. Moreover, excessive use of antimicrobials may increase bacterial resistance, potentially leading to secondary infections. Currently, laser technology is becoming widely adopted in oral disease treatment, this approach provides several advantages including high patient acceptance, ease of use, minimally invasive application, tissue hemostasis and accelerated healing. In addition, lasers are especially effective at removing granulation tissue from implant surfaces without causing melting, cracking or deformation (Shibli, 2018). Unlike natural roots or surrounding biological tissues, the effectiveness of different laser types with varying wavelengths may differ when used on titanium surfaces. Studies have indicated that CO_2_ and diode lasers are not effective in removing plaque biofilms from root surfaces or titanium implants. These types of lasers have only been used in addition to mechanical treatment procedures (Chen et al., 2022a). In contrast, the Er laser has shown significant bactericidal potential in periodontal treatment, effectively eliminating bacterial contaminants from the textured surfaces of implants (Cosgarea et al., 2023; Aoki et al., 2024). The Er: YAG laser directly impairs bacterial viability by disrupting LPS and reducing anaerobic bacterial counts (Chen et al., 2022a), and it also mitigates inflammatory damage by regulating the miR-155/SIRT1 axis and the activation of IL-6 and NF-κB signaling pathways, thereby affecting osteogenic differentiation, inflammation, proliferation, apoptosis and autophagy (Ogita et al., 2015; Lin et al., 2021; Ng et al., 2024) — ultimately preserving tissue and minimizing damage. Antimicrobial photodynamic therapy (aPDT) employs laser and photosensitizer at specific wavelengths to generate ROS, inducing superoxide dismutation reaction that disrupts bacterial lipid layers. ROS penetrate bacterial lipid membranes, leading to protein leakage and selectively eliminating anaerobes and their metabolites. The above therapies have been shown to significantly reduce *P. intermedia/nigrescens*, *Fusobacterium* spp., and beta-hemolytic *Streptococcus*, even decreasing plaque accumulation by up to 70% (Shibli et al., 2003; Mao et al., 2019).

Dietary component nitrate has garnered significant attention for its critical role in maintaining periodontal tissue health. This importance is highlighted by its interactions with the oral microbiota, its capacity to regulate inflammatory responses, and its promising potential as an adjunctive therapeutic option for managing periodontal disease (Rosier et al., 2022). Recent studies demonstrate that nitrate serves as a substrate for nitrate-reducing bacteria, with *Rothia* serving as the predominant genus in this process, including *Veillonella* and *Actinomyces*, which enzymatically convert NO_3_^−^ into nitrite NO_2_^−^. Nitrite subsequently acts as a precursor for NO, a critical signaling molecule with diverse biological effects (Wicaksono et al., 2020; Schlagenhauf, 2022). NO exerts substantial antimicrobial activity by inhibiting the key periodontal pathogens such as *P. gingivalis* and *F. nucleatum*. It also modulates the abundance of approximately 20 microbial species, including notable genera such as *Campylobacter, Fusobacterium, Neisseria, Prevotella, Rothia, Selenomonas, Staphylococcus*, *Streptococcus* and others. This microbial regulation helps maintain oral microbial homeostasis and reduces dysbiosis within periodontal pockets (L’Heureux et al., 2023). In addition, NO exhibits robust immunomodulatory properties, suppressing the production of pro-inflammatory cytokines to prevent excessive immune activation. This modulation reduces inflammatory burden, thereby protecting against periodontal tissue destruction. Furthermore, NO regulates critical pathways such as NF-κB signaling and ROS production, facilitating a balance between protective inflammation and tissue damage. Additionally, it enhances endothelial cell function and angiogenesis, improving vascular perfusion and promoting tissue repair — essential processes for periodontal regeneration (Rammos et al., 2015; Yang et al., 2020b). Nitrate-derived NO also synergizes with antimicrobial therapies, restoring microbial balance and supporting long-term periodontal stability. Integrating nitrate-based strategies into periodontal treatment paradigms represents an innovative approach, with nitrate supplementation showing potential as an adjunctive therapy for periodontal disease. Future explorations should focus on understanding nitrate bioavailability and microbial dynamics. Personalized strategies for optimizing nitrate metabolism are also essential to fully harness its therapeutic potential in periodontal disease management and oral health maintenance (Rosier et al., 2020). While excessive local application of nitrate (at concentrations exceeding 100 nM) may exhibit toxic effects on oral microbiota, its application as a prebiotic, either alone or in conjunction with nitrate-reducing probiotics, represents a promising adjunctive approach for the treatment of periodontitis (Wicaksono et al., 2020; Mazurel et al., 2023).

## 4 Conclusion

Bacterial dysbiosis is considered one of the central causes driving the development of periodontitis and peri-implantitis. The proliferation of pathogenic bacteria, biofilm formation and dysregulation of systemic immune responses collectively contribute to the onset and progression of these inflammatory diseases. To maintain the delicate balance between bone resorption and regeneration, the osteoclast-osteoblast equilibrium must be properly regulated to ensure effective alveolar bone remodeling and homeostasis. During bacterial infections, the inflammasome activation could induce bacterial dissemination and uncontrolled bone destruction. These conditions are commonly seen in periodontitis, periapical inflammation, peri-implantitis and other related diseases, all of which share similar mechanisms. Uncontrolled inflammasome activity also enhances the activation of macrophages, monocytes, neutrophils and other adaptive immune cells such as Th17 cells, resulting in increased osteoclast numbers and suppressed osteoblast activity, ultimately eventuating alveolar bone loss. Additionally, osteocytes play a key role in this process by secreting many cytokines that regulate alveolar bone resorption and formation in response to inflammatory signal changes (Huang et al., 2020).

The peri-implant microbiota exhibits notable differences from the periodontal niche, including lower diversity. Changes in microbial community composition are inextricably linked to transitions between health and disease, with host-microbiota interactions may contributing to the development of peri-implantitis. Insights into the specificity of the oral microbiome underscore the complexity and challenges of managing oral infections. This review discussed the differences in common microbial species between healthy periodontal and inflamed regions, as well as their pathogenic mechanisms. Understanding the mechanisms of changes in bacterial flora is of great significance for the prevention, diagnosis and treatment of oral diseases. This review suggests that dysbiosis in the oral microbiome may act as the initial disturbance in bacterial species balance, with pathogenic bacteria gaining dominance by forming robust biofilms, which directly or indirectly intensify tissue destruction. Inflammation often progresses in a non-linear increasing pattern and is frequently accompanied by significant crestal bone loss. Plaque control through routine maintenance and early intervention helps retain a healthy oral environment and significantly reduces the risk of periodontal inflammation. Besides, further research is needed to clarify the mechanisms underlying the transition from peri-implant mucositis to peri-implantitis and to optimize current prevention and treatment strategies.
